# A feature fusion deep-projection convolution neural network for vehicle detection in aerial images

**DOI:** 10.1371/journal.pone.0250782

**Published:** 2021-05-07

**Authors:** Bin Wang, Bin Xu

**Affiliations:** 1 School of Communication and Information Engineering, Shanghai University, Shanghai, China; 2 Shanghai Institute for Advanced Communication and Data Science, Shanghai University, Shanghai, China; Norfolk State University, UNITED STATES

## Abstract

With the rapid development of Unmanned Aerial Vehicles, vehicle detection in aerial images plays an important role in different applications. Comparing with general object detection problems, vehicle detection in aerial images is still a challenging research topic since it is plagued by various unique factors, e.g. different camera angle, small vehicle size and complex background. In this paper, a Feature Fusion Deep-Projection Convolution Neural Network is proposed to enhance the ability to detect small vehicles in aerial images. The backbone of the proposed framework utilizes a novel residual block named stepwise res-block to explore high-level semantic features as well as conserve low-level detail features at the same time. A specially designed feature fusion module is adopted in the proposed framework to further balance the features obtained from different levels of the backbone. A deep-projection deconvolution module is used to minimize the impact of the information contamination introduced by down-sampling/up-sampling processes. The proposed framework has been evaluated by UCAS-AOD, VEDAI, and DOTA datasets. According to the evaluation results, the proposed framework outperforms other state-of-the-art vehicle detection algorithms for aerial images.

## 1. Introduction

As one of the core research topic of computer vision, object detection is widely used in automatic driving, crowd flow counting, topographic exploration, environmental pollution monitoring, etc. The task of object detection is to find out the various targets in the images, and determine the locations and categories of these targets. Because the appearance of objects changes significantly according to various factors [1–4], object detection is commonly regarded as one of the most challenging tasks in the field of computer vision.

The traditional object detection algorithms are normally based on hand-crafted features or textures. R.M. Haralick et al. proposed textural features for image classification in 1973 [5]. D. G. Lowe proposed scale-invariant feature transform (SIFT) in 1999 [6]. In 2014, T. Moranduzzo and F. Melgani used SIFT to count the number of vehicles and trained a support vector machine (SVM) to classify vehicle and non-vehicle objects based on the SIFT descriptors [7]. S. Qi et al. proposed a ship detector for optical satellite images based on saliency and S-HOG descriptor in 2015 [8]. In recent years, due to the significant development of the neural network, object detection algorithms based on convolutional neural network(CNN) outperform traditional object detection algorithms in almost every aspect [9, 10]. According to [11, 12], object detection algorithms based on CNN could be roughly divided into two categories: two-stage and one-stage object detection algorithms. Two-stage object detection algorithms consist of two models. The first model is designed for distinguishing interesting targets from the background; the second model is responsible for classifying these interesting targets into various categories. Typical two-stage object detection algorithms are Faster R-CNN [13], Mask R-CNN [14], and so on. One-stage object detection algorithms integrate the region proposal model and the classification network into a single neural network to improve feature reusability and detection speed. Typical one-stage object detection algorithms are YOLOv3 [15], Single-Shot Multibox Detector (SSD) [16], RetinaNet [17] and so on.

Vehicle detection in aerial images, as an important branch of object detection, has been intensively researched recently. Along with the development of Unmanned Aerial Vehicles(UAVs), vehicle detection in aerial images plays an important role in various kinds of fields. Comparing with general object detection problems, vehicle detection in aerial images has its unique characteristics. According to [3, 18–21], the main difficulties of object detection in aerial images are as follows:

- The particularity of perspective. Aerial images are usually taken from the top of the sky, but most images of the conventional datasets were shot from the horizontal perspective at the ground. Therefore, the patterns of the objects are different in aerial images comparing with the same objects in conventional datasets. From the top view, the objects usually contain fewer texture features, and it is easier to confuse the targets with other objects.
- The size of the objects. Comparing to the objects in images taken on the ground, the objects in aerial images are generally small (composed by only dozens or even fewer pixels), thus these objects contain only a little information. Furthermore, the down-sampling layers in CNN will reduce the amount of information contained in each object, e.g. after four down-sampling layers, a 24 * 24 object retains only about one pixel in feature maps, which makes it difficult to distinguish small objects from the background.
- The complexity of the background. The receptive field of aerial images is relatively large (covering an area of several square kilometers). This receptive field may contain various backgrounds, e.g. farmland, mountains, urban area and so on, which will lead to strong and diversified interference to the object detection process.

In general, object detection in aerial images requires detecting small objects at specific angles under complex backgrounds. As a result, making full use of limited information is the key to accurate detection. The low-level features contain more detailed information, such as edges, corners, lines, color, and so on, while the high-level information contains more abstract semantic information. To get high-level semantic features, the input images need to be processed by a large number of convolutional layers, which will blur the low-level features. For small object detection, it even causes the loss of key features of objects, which usually leads to detection failures. Therefore, the state-of-the-art general object detectors usually do not perform well when processing aerial images.

Considering the unique requirements of vehicle detection in aerial images, a Feature Fusion Deep-Projection Convolution Neural Network (FFDP-CNN) is proposed in this paper. The main contributions of this paper are as follow:

1. A novel residual block named stepwise res-block is proposed. To explore high-level semantic features, a single stepwise res-block contains three 3x3 convolutional layers. These three layers form a hierarchical structure that produces features processed by various numbers of convolution layers. Comparing to the original res-block, the proposed stepwise res-block increases the depth of the network, and keeps the parameter number at a relatively low level. In addition to the shortcut connection adding to the output, the proposed stepwise res-block introduces another shortcut to conserve the low-level features.
2. Based on the proposed stepwise res-block, a backbone for small object detection is designed. This backbone is composed of 34 stepwise res-blocks. Comparing with the original ResNet or Res2Net with roughly the same parameter number, the proposed backbone provides more convolutional layers which enhance its capacities of extracting complex high-level semantic features. Meanwhile, the combination of multiple stepwise res-block enables the output features to be processed by various numbers of convolutional layers, as well as conserves the low-level features which are important in small object detection. Thus the feature generated by the proposed backbone contains both low-level detailed information and high-level semantic information at the same time.
3. A new feature fusion and detection network is proposed. To make full use of the high-level features and low-level features generated by the backbone, a feature fusion and multi-scale detection network is designed. The outputs from different levels of the proposed backbone are collected into the feature library through the spatial pyramid structure, and the features suitable for detection are obtained through an automatic selection process of the network. The selected features are then fed into the multi-scale detection module for detection. To reduce the information contamination introduced by up-sampling/down-sampling processes, a deep-projection deconvolution module is adopted in the proposed feature fusion network.

The rest of this paper is organized as follows. Section 2 reviews the state-of-the-art vehicle detection algorithms for aerial images; The implementation detail of the proposed Feature Fusion Deep-Projection Convolution Neural Network is described in section 3; Section 4 contains the evaluation results of the proposed framework using three different datasets; section 5 concludes this paper.

## 2. Related work

Due to the significant performance advantages of Convolution Neural Network (CNN), the most recent proposed vehicle detection algorithms for aerial images are based on various CNNs. In 2017, T. Tang et al. designed an improved Faster-RCNN to solve the difficulties of locating the positions of small vehicles and classifying the vehicle from the background [22]. The authors use a hyper region proposal network (HRPN) combined with different levels of feature maps to extract vehicles, and an enhanced classifier to reduce false detection.

In 2018, S. Liu et al. proposed a Path Aggregation Network (PANet) which aims at solving the problem of boosting information flow in a proposal-based instance segmentation framework [23]. By extending the bottom-up path, an accurate positioning signal can be obtained at the bottom layer. Thereby the entire feature hierarchy is enhanced, and the information path between the bottom layer and the topmost feature is shortened. The authors also proposed an adaptive feature pool to link all features, so that the useful information of each layer can be directly transmitted to the proposal subnetwork.

M.Y. Yang et al. presented a novel double focal loss convolutional neural network framework (DFL-CNN) in 2018 [24]. The proposed framework uses skip connection and focal loss functions to improve detection performance. At the same time, a new large-scale vehicle detection dataset named ITCVD was proposed in the same paper. According to the authors, DFL-CNN obtains good performance on the ITCVD dataset.

In 2018, Y. Koga et al applied Hard Example Mining (HEM) to Stochastic Gradient Descent (SGD) in the process of training neural networks [12]. According to the authors, the application of HEM can improve detection accuracy by training the networks with more informative samples.

G. Cheng et al tried to solve the detecting problem related to object rotation, within-class diversity and inter-class similarity by adding a rotation-invariant regularizer and a fisher discrimination regularizer to the existing neural networks in 2018 [25]. Compared with the other state-of-the-art algorithms, the authors claimed that their method achieved good performance on various datasets.

In 2019, Murari Mandal et al. designed a one-stage vehicle detection network (AVDNet) [11]. The proposed network used ConvRes to hold the features of small objects on multiple scales. The author also proposed a recurrent-feature aware visualization (RFAV) technique to analyze the layers in the network.

W. Liu et al. also introduced an object detection algorithm for aerial images in 2019 [26]. A feature introducing strategy based on oriented response dilated convolution to make the model adaptable to multiscale objection detection.

Qiu H et al. proposed a novel end-to-end Adaptively Aspect Ratio multi-scale Network (A2RMNet) which focuses on detecting objects with various sizes and aspect ratios in 2019 [27]. The authors designed a feature gate fusion network to integrate multi-scale feature maps, and an aspect ratio attention network to prevent the changes of objects’ aspect ratios.

Artacho, B et al. proposed a new semantic segmentation network in 2019 [28]. The main structure of the network is named "Waterfall" Atrous Spatial Pooling (WASP) architecture. Compared with the traditional spatial pyramid structure, WASP can reduce the number of parameters used in the network, while increasing the receptive field of the network.

W.Li et al. proposed a network to detect and count vehicles simultaneously in aerial images in 2019 [4]. The authors utilized the combination of bottom-up cues and top-down attention mechanisms to maximize the use of mutual information between object categories and features. An effective loss function is used in the proposed network to promote the ability to push the anchors toward matching the ground-truth boxes as much as possible.

In 2020, Y. Yang et al. proposed a framework to solve the perspective distortion problem of aerial images [29]. The proposed framework utilized a reverse perspective network to evaluate perspective distortion, and evenly distort the image to obtain similar example scales. At the same time, the framework forces the regressor to learn from the augmented density maps via an adversarial network to further solve the problem of scale distortion in dense regions.

Rabbi. J et al. proposed a novel small object detection framework by combining a generative adversarial network (GAN)-based model called enhanced super-resolution GAN (ESRGAN), an Edge-Enhancement Network EEN and a detection network in 2020 [30]. The proposed framework achieved promising performance on both public datasets and self-assembled dataset.

A brief summary of some related works and their contributions is shown in Table 1.

**Table 1:** The summarize of the significant related work and contributions.

Algorithms	Contribution
AVDNet 2019 [11]	1. Introducing ConvRes residual blocks at multiple scales to alleviate the problem of vanishing features for smaller objects caused
	2. Proposing a recurrent-feature aware visualization (RFAV) technique to analyze the network behavior.
	3. A new airborne image data set (ABD)
W. Liu et al. 2019 [26]	1. Concatenating feature maps from layers of different depths
	2. Adopting a feature introducing strategy based on oriented response dilated convolution.
A2RMNet 2019 [27]	1. A multi-scale feature gate fusion network which is composed of gate fusion modules, refine blocks and region proposal networks.
	2. An aspect ratio attention network is leveraged to preserve the aspect ratios of objects
Artacho, B et al. 2019 [28]	1. A new efficient architecture for semantic segmentation, based on a “Waterfall” Atrous Spatial Pooling architecture, that achieves a considerable accuracy increase while decreasing the number of network parameters and memory footprint.
W.Li et al. 2019 [4]	1. Exploring why the state-of-the-art detectors fail in highly dense drone scenes
	2. An effective loss
	3. Combining bottom-up cues with top-down attention mechanisms
Rabbi. J et al. 2020 [30]	1. A new edge-enhanced super-resolution GAN (EESRGAN) is applied to improve the quality of remote sensing images
	2. Using different detector networks in an end-to-end manner where detector loss was back-propagated into the EESRGAN to improve the detection performance.

## 3. Method

### 3.1. Introduction of proposed framework

**Fig 1:**
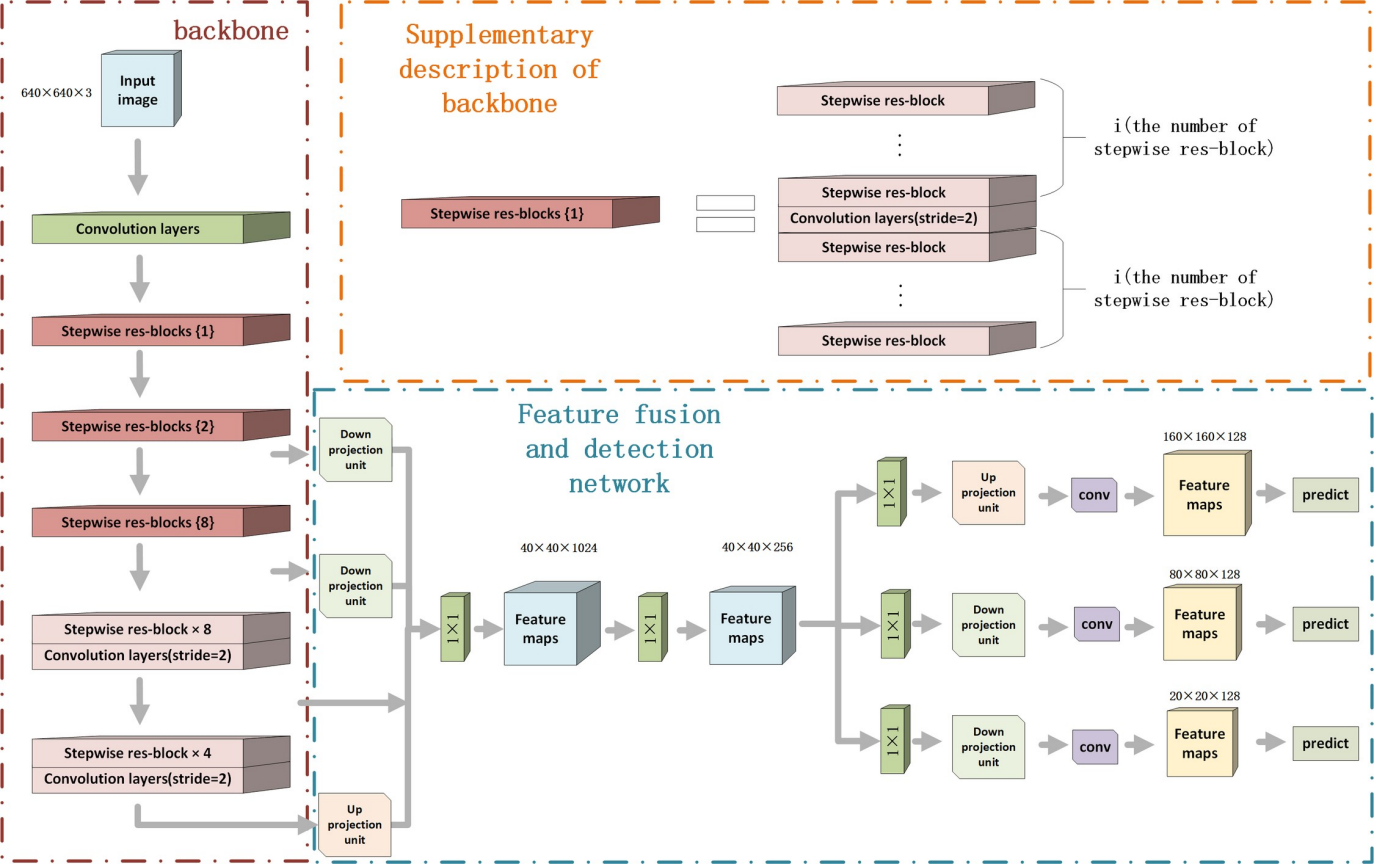
Overview of the proposed framework.

As shown in Fig 1, the proposed Feature Fusion Deep-Projection Convolution Neural Network (FFDP-CNN) can be roughly divided into two parts: a backbone network composed of stepwise res-blocks, and the feature fusion network incorporating with the detection network. Thanks to the stepwise res-block, the proposed backbone produces features processed by various numbers of convolution layers, which helps to conserve low-level detailed information and produce high-level semantic information. The feature fusion network fuses the low-level and high-level feature maps and selects the information that is effective to vehicle detection in the different feature maps.

### 3.2. Introduction of stepwise res-block

The original residual block [31] is proposed to solve the vanishing gradient problem which appears as the depth of the network increases. By stacking up multiple residual blocks, networks with various depth have been proposed to better explore high-level semantic features. However, in small object detection, low-level detailed information is considered as important as high-level semantic information. Although the side connection in the original residual block helps to increase the depth of the network, it cannot prevent the disappear of low-level detailed information caused by convolutions. To obtain the low-level detailed information and high-level semantic information at the same time, a stepwise hierarchical convolution structure is created to replace the 3×3 convolution layer in the original residual block. Because the input feature is divided and processed hierarchically, like climbing stairs, the proposed res-block is named as stepwise res-block.

**Fig 2:**
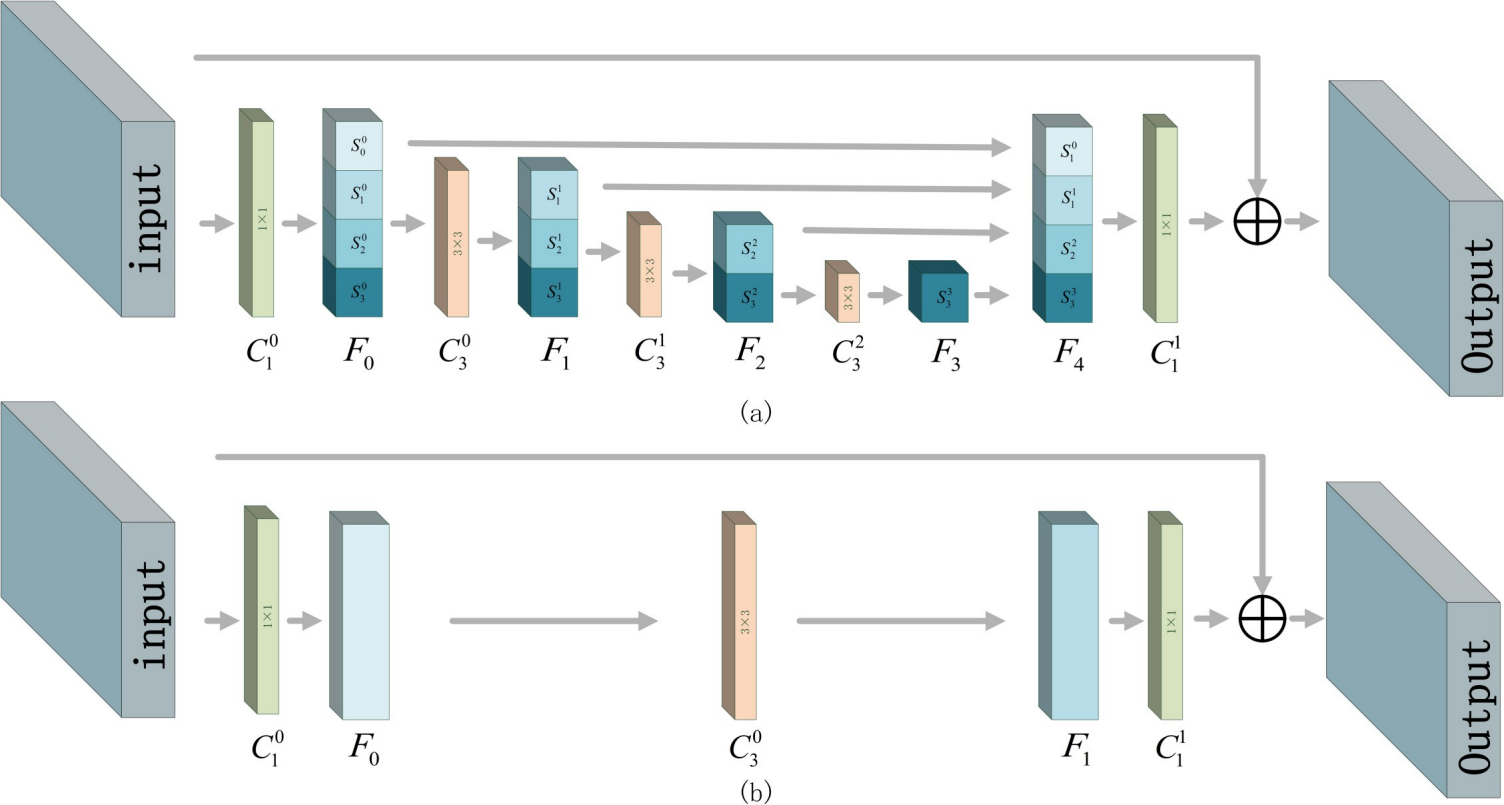
Comparison between stepwise res-block and res-block, (a) is stepwise res-block, (b) is res-block.

The structure of the proposed stepwise res-block is shown in Fig 2. $S_{j}^{i}$ indicates a feature subset (i indicates the number of convolution layers the subset has been processed by, i∈ (0,3), j indicates the subset number, j∈ (0,3)). *F*_*i*_ indicates a feature map assembled by feature subsets(i indicate the number of this feature map). $C_{i}^{j}$ indicates a i×i convolution layer. The input of stepwise res-block is first processed by a $C_{1}^{0}$ to decrease the channel number (to 1/4 of the input’s original channel number), and then split into even quarters ($S_{0}^{0},\;S_{1}^{0},\;S_{2}^{0},\;S_{3}^{0}$) by the number of channel. $S_{0}^{0}$ is directly superimposed onto *F*_4_ without processing, while $S_{1}^{0},\;S_{2}^{0},\;S_{3}^{0}$ pass through $C_{3}^{0}$ to obtain $S_{1}^{1},\;S_{2}^{1},\;S_{3}^{1}$ (collectively called *F*_1_). Then, $S_{1}^{1}$ is directly superimposed onto *F*_4_ without processing, while $S_{2}^{1},\;S_{3}^{1}$ pass through $C_{3}^{1}$ to obtain $S_{2}^{2},\;S_{3}^{2}$ (collectively called *F*_2_). After that, $S_{2}^{2}$ is directly superimposed onto *F*_4_ without processing, while $S_{3}^{2}$ passes through $C_{3}^{2}$ to obtain $S_{3}^{3}$ (also named *F*_2_) which is superimposed onto *F*_4_. It can be seen that $S_{0}^{0},\;S_{1}^{1},\;S_{2}^{2}$ and $S_{3}^{3}$ are concatenated together to form feature map *F*_4_. At the end of this process, *F*_4_ is processed by another 1×1 convolution layer $C_{1}^{1}$ to restore the channel number to the input’s original channel number. Side connection is also used in stepwise res-block after *F*_4_ passing through a $C_{1}^{1}$.

Different subsets of output processed by different numbers of convolution layers, resulting in different subsets containing features with different receptive fields. Among them, small receptive field subsets, such as $S_{0}^{0},\;S_{1}^{1}$, pass through convolution layers fewer times, contain more detail information which is important for small object detection. Subsets with large receptive field, such as $S_{2}^{2},\;S_{3}^{3}$, blur the detail information but can explore deep semantic information which is also important for detection [21]. Two *C*_1_ are used at the beginning and end of the proposed block to automatically select the suitable features and implement a bottleneck to reduce the parameter number.

Comparing with the original res-block, stepwise res-block can provide more maximum depth with fewer parameters. Assuming that the dimension of the input and the outputs is n*n*m, the comparison of parameter number between the original res-block and stepwise res-block is shown in Table 2.

**Table 2:** Parameter comparison between original res-block and stepwise res-block.

Block name	Original res-block	Stepwise res-block
Parameter of $C_{1}^{0}$	1×1×m	1×1×m
Parameter of $C_{3}^{0}$	3×3×m	3×3×m×$\frac{3}{4}$
Parameter of $C_{3}^{1}$	-	3×3×m×$\frac{3}{4}$ × $\frac{2}{3}$
Parameter of $C_{3}^{2}$	-	3×3×m×$\frac{3}{4}$ × $\frac{2}{3}$ × $\frac{1}{2}$
Parameter of $C_{1}^{1}$	1×1×m	1×1×m
Total number of parameter	3×3×m×$\frac{5}{3}$	3×3×m×$\frac{13}{6}$
Max convolution Number (ignoring *C*_1_)	1	3
Average parameter per convolution	3×3×m×$\frac{30}{18}$	3×3×m×$\frac{13}{18}$

Compared with the original residual module (as shown in Table 2), the stepwise res-block can add two more convolution layer to the network (ignoring the 1 x 1 convolution layers), but the number of parameters has increased by only 30%, which is more suitable for composing deep network and exploring the underlying semantic information. The effectiveness and efficiency of the proposed stepwise res-block are validated in the experiments shown in section 4.4.

### 3.3. Introduction of backbone based on stepwise res-block

The stepwise res-block can be used in the mainstream networks to replace the convolution layer or the residual module, but to fully explore its advantages, a backbone based on the proposed stepwise res-block is designed in this section. The network structure is shown in Table 3.

**Table 3:** The structure of backbone.

	Type	Filters	Size/Stride	Output
	Stepwise res-block	32	3×3/1	640×640
	Convolution layer	32	3×3/2	320×320
2×	Stepwise res-block	64	3×3/1	320×320
	Convolution layer	64	3×3/2	160×160
2×	Stepwise res-block	64	3×3/1	160×160
8×	Stepwise res-block	128	3×3/1	160×160
	Convolution layer	128	3×3/2	80×80
8×	Stepwise res-block	128	3×3/1	80×80
8×	Stepwise res-block	256	3×3/1	80×80
	Convolution layer	256	3×3/2	40×40
4×	Stepwise res-block	256	3×3/1	40×40
	Convolution layer	256	3×3/2	20×20

Taking full advantage of stepwise res-block, the proposed backbone can explore high-level semantic information and conserve low-level details information at the same time. To explore high-level semantic information, 34 stepwise res-blocks (each stepwise res-block contains three 3x3 convolutional layers) is used in the proposed backbone. As a result, the maximum depth of the proposed backbone is 102 3x3 convolutional layers. As a comparison, widely used ResNet101 only contains 33 3x3 convolutional layers. A deeper network can generate better nonlinear expressions and semantic features. At the same time, the task of each convolutional layer becomes clearer while the depth of the networks goes deeper [21].

On the other hand, thanks to the stepwise res-block, the output of the proposed backbone has hierarchical receptive fields. Given the fact that the amount of input processed by 0–3 convolutional layers are divided equally in each stepwise res-block, the output of the proposed backbone contains feature generated by various numbers of convolutional layers (0–102, distributed equally if 1x1 convolutional layers are ignored), which increase the diversity of the features contained in the output. As a result, as well as high-level semantic features, there are plentiful low-level or mid-level features in the output.

### 3.4. Introduction of feature fusion deep-projection module and multi-scale detection network

**Fig 3:**
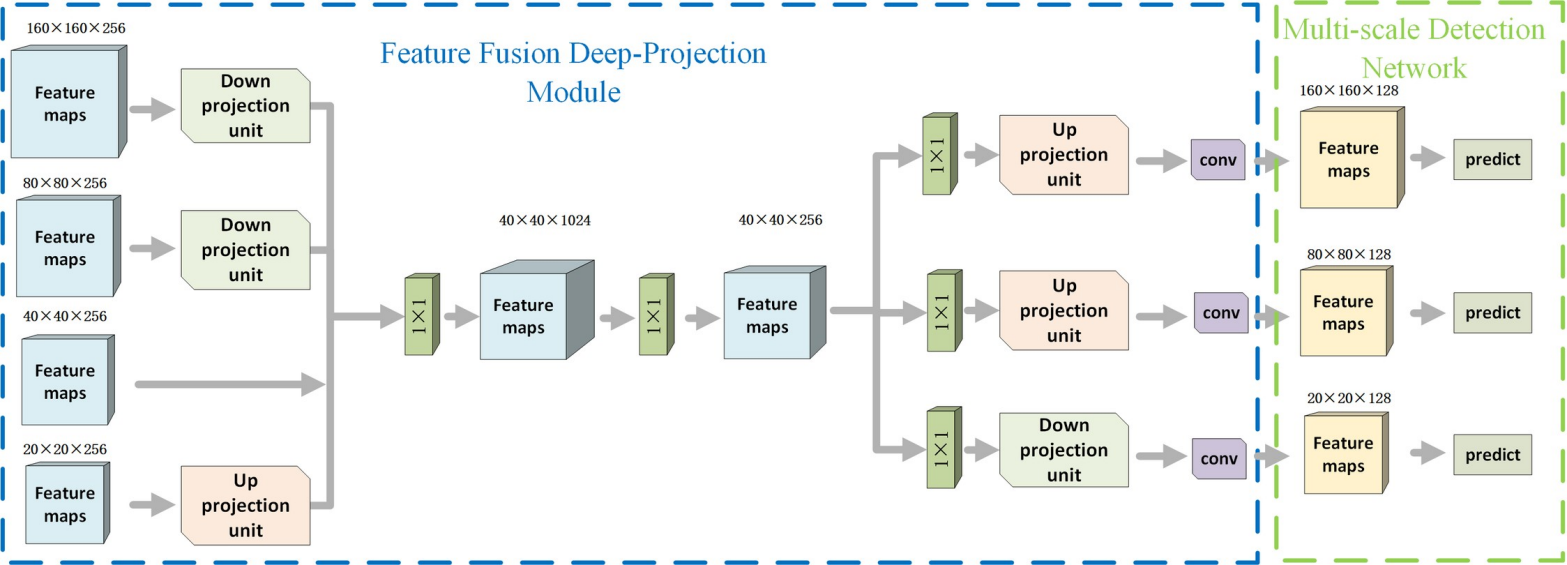
Feature fusion and multi-scale detection network.

Since the feature maps obtained from different levels of the proposed backbone have different dimensions, a feature fusion module is required to mix features generated by different levels of the proposed backbone together to ensure each detector receives feature maps containing full information of targets. To avoid feature loss, deep-projection deconvolution is used in the feature maps down/up-sampling process. As shown in Fig 3, feature maps with different scales go through the feature fusion deep-projection module and multi-scale detection network to generate detection results. The feature fusion deep-projection module is responsible for mixing Feature images of different levels and restoring them to the specified resolution. On the one hand, low-level and high-level features can be fused together to generate feature maps containing both semantic and detail information for the detectors. On the other hand, the deep-projection deconvolution module implemented in the down/up projection unit prevents the information contamination introduced by up-sampling/down-sampling steps. The detailed process of the proposed feature fusion deep-projection module is represented in section 3.4.1. The multi-scale detection network of the proposed framework remains the same as YOLO V3, while the loss function is changed to mine hard samples. The detailed introduction of the multi-scale detection network can be found in section 3.4.2.

#### Introduction of feature fusion deep-projection module

In general, the output from different levels of the proposed backbone is sampled and concatenated into a collection of feature maps. Multiple 1×1 convolutions are implemented before and after the obtained collection of feature maps to implement a feature selection process. The selected feature is then restored to feature maps with different resolutions which finally sent to various detectors. Because of the proposed feature fusion network, each detector can use both the high-level and low-level information from the backbone.

**Fig 4:**
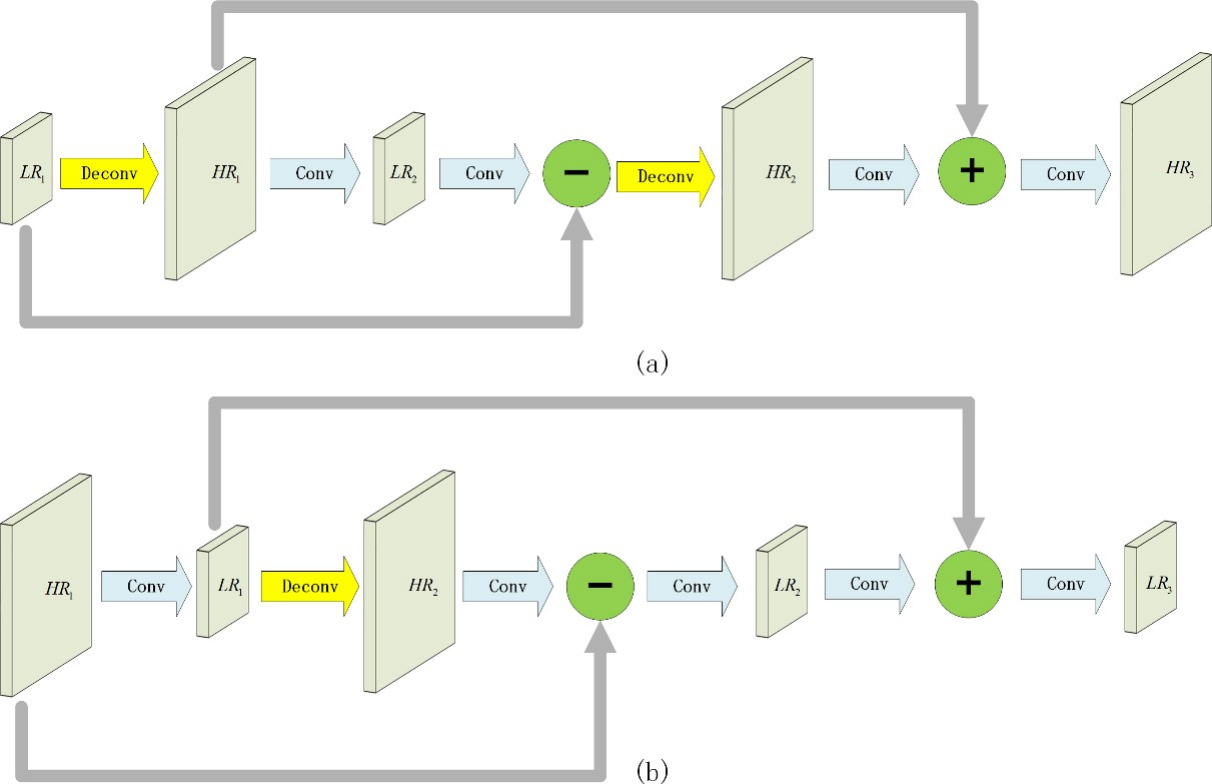
Deep-projection deconvolution model. (a) is up projection unit. (b) is down projection unit.

Because of the down-sampling and up-sampling steps used in the proposed feature fusion network, information contamination is unavoidable. To reduce the impact of information contamination, inspired by the deep-projection unit used in super-resolution image reconstruction algorithms [32], a deep-projection deconvolution module is implemented in the proposed feature fusion network. The detailed structure of the proposed deep-projection deconvolution module is shown in Fig 4. It contains two different components: the up projection unit (for up-sampling) and the down projection unit (for down-sampling). In the up projection unit, as shown in Fig 4(A), the low-resolution feature maps *LR*_1_ go through a deconvolution layer to get the high-resolution feature maps *HR*_1_ firstly. Then the high-resolution feature maps *HR*_1_ pass through a convolution layer to obtain a low-resolution feature maps *LR*_2_. The residual result of the obtained low-resolution feature maps *LR*_2_ and the initial low-resolution feature maps *LR*_1_ goes through another deconvolution layer to obtain high-resolution feature maps *HR*_2_. Finally, the high-resolution feature maps *HR*_2_ (after passing through a convolution layer) and the high-resolution feature maps *HR*_1_ obtained initially are added together to obtain the final high-resolution feature map *HR*_3_. In the down projection unit, as shown in Fig 4(B), the high-resolution feature maps *HR*_1_ go through convolution layer to get the low-resolution feature maps *LR*_1_ firstly. Then the low-resolution *LR*_1_ passes through a deconvolution layer to obtain the high-resolution feature maps *HR*_2_. The residual result of the obtained high-resolution feature maps *HR*_2_ and the initial high-resolution feature maps *HR*_1_ goes through a convolution layer to obtain the low-resolution feature maps *LR*_2_. Finally, the low-resolution feature maps *LR*_2_ (after passing through a convolution layer) and the low-resolution feature maps *LR*_1_ obtained initially are added together to obtain the final low-resolution feature map *LR*_3_. These projection units continuously correct themselves and produce accurate feature maps which will be fed into 3 detectors later. The effectiveness of the proposed feature fusion deep-projection module is validated in the experiments shown in section 4.4.

#### 3.4.2. Introduction of improved multi-scale detection network

**Fig 5:**
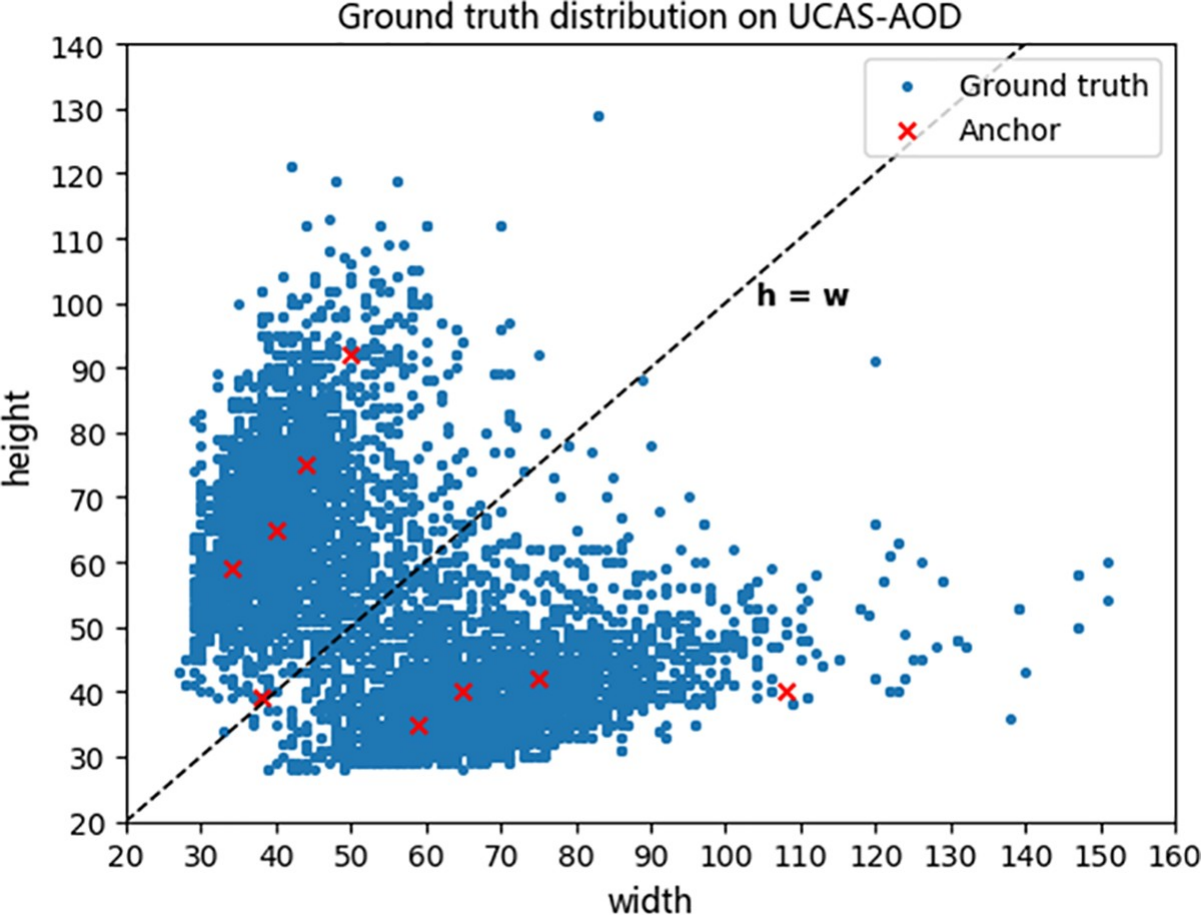
The distribution of ground truth on UCAS-AOD.

The detection network used in the proposed framework is improved from the YOLOv3 framework. The anchor boxes used in the proposed detection network are optimized according to the target sizes of the evaluation datasets since the original anchor boxes used in YOLOv3 are obtained by clustering the target sizes in the COCO dataset and not suitable for vehicle detection in aerial images. Multi-scale detection is kept in the proposed detection network. Each scale uses different anchors, which is shown in Fig 5, and is responsible for detecting different groups of targets. The 80×80 scale detector, which is responsible for the mainstream targets detection tasks, is assigned to anchors with medium sizes, e.g. (65×40), (44×75), (40×65). The 20×20 scale detector, which is responsible for the relatively large targets detection tasks, is assigned to anchors with relatively large size, e.g. (75×42), (50×92), (108×40). The 160×160 scale detector, which is responsible for relatively small targets detection tasks, is assigned to anchors with relatively small size, e.g. (38×39), (34×59), (59×35).

The loss function is used to compute the error between the predicted value and true value. The loss function in our framework is improved from the loss function used in YOLOv3. The original loss function is consist of three parts: location prediction box loss, IOU loss, classification loss. The detailed description is introduced in [33].

$$Loss=Loss_{location}+Loss_{class}+Loss_{confidence},$$ (1)

$$\begin{array}{l} {\mathrm{Loss}}_{\mathrm{location}}=\lambda_{\mathrm{coord}}\sum_{i=0}^{s^{2}}\sum_{j=0}^{B}l_{ij}^{obj}\left[{\left(x_{i}-{\hat{x}}_{i}\right)}^{2}+{\left(y_{i}-{\hat{y}}_{i}\right)}^{2}\right]\\ +\lambda_{\mathrm{coord}}\sum_{i=0}^{s^{2}}\sum_{j=0}^{B}l_{ij}^{obj}\left[{\left(w_{i}-{\hat{w}}_{i}\right)}^{2}+{\left(h_{i}-{\hat{h}}_{i}\right)}^{2}\right], \end{array}$$ (2)

$$Loss_{class}=\sum_{i=0}^{s^{2}}l_{ij}^{obj}\sum_{j=0}^{B}\left[{\left(p_{i}\left(c\right)-{\hat{p}}_{i}\left(c\right)\right)}^{2}\right],$$ (3)

$$\begin{array}{l} {\mathrm{Loss}}_{IOU}=\sum_{i=0}^{s^{2}}\sum_{j=0}^{B}l_{ij}^{obj}\left[\left(c_{i}-c_{i}\right)\right]\\ +\lambda_{\mathrm{noord}}\sum_{i=0}^{s^{2}}\sum_{j=0}^{B}l_{ij}^{noobj}\left[{\left(c_{i}-c_{i}\right)}^{2}\right], \end{array}$$ (4)

*λ*_*coord*_, *λ*_*noord*_ represent the weight of the corresponding loss, *s*^2^ represents the total number of grids in the image, B represents the total number of predicted bounding box in each grid, $l_{ij}^{obj}$ = 1 represents that the *j*th predicted bounding box is effective detection in *i*th grid. *λ*_*coord*_, *λ*_*noord*_ is assigned 1 in our network.

Since one stage network without region proposal network (RPN) is usually plagued by overwhelming negative samples, a hard sample mining strategy is added in our classification loss. The improved classification loss is shown below.

$$Loss_{class}=M_{HSM}\sum_{i=0}^{s^{2}}l_{ij}^{obj}\sum_{j=0}^{B}\left[{\left(p_{i}\left(c\right)-{\hat{p}}_{i}\left(c\right)\right)}^{2}\right],$$ (5)

$$M_{HSM}\left(P_{i}\left(c\right)\right)=\{\begin{array}{c} 10^{-x}\;P_{i}\left(c\right)>\alpha \\ -\log\left(1-x\right)\;P_{i}\left(c\right)\leq \alpha \end{array},$$ (6)

α represents a constant that is usually equal to the score of vehicle determination. When a sample is an emblematic negative sample, *P*_*i*_(*c*) is close to 0, and this emblematic negative sample leads to a small effect on the back propagation process. When a sample is a hard sample, *P*_*i*_(*c*) is close to the score of vehicle determination. $M_{HSM}$ obtains a high value, which improves the effect on the back propagation process. Therefore, the hard sample mining strategy filters the impact of a large number of negative samples on classification, and at the same time strengthens the impact of hard samples on classification.

## 4. Experiments and results

### 4.1. Dataset

#### 4.1.1. Brief introduction

The increase of datasets has been one of the driving forces for the rapid development of object detection algorithms in recent years. Commonly used datasets, such as VOC [34] and COCO [35], not only provide data for various algorithms, but also a baseline for performance comparisons.

Comparing with the images contained in datasets designed for general object detection or image classification, aerial images have special characteristics. It is unlikely to train vehicle detection algorithms for aerial images only based on datasets designed for general object detection algorithms or image classification algorithms. As a result, several aerial images datasets, e.g. RSOD [36], INRIA [37], UCAS-AOD [19], VEDAI [18], DOTA [20] are announced as vehicle detection in aerial images has developed significantly recently. In this paper, three commonly used datasets designed for vehicle detection in aerial images, i.e. UCAS-AOD, VEDAI and DOTA, are used in the evaluation section. Since Images form theses three datasets are taken by UAVs or satellites from high altitudes, the sizes of the targets in these three datasets vary while the imaging angle of these targets remains the same.

#### 4.1.2. UCAS-AOD datasets

The UCAS-AOD Dataset is proposed in [19] in 2015. The images contained in UCAS-AOD are selected from Google earth, and labelled by Patterns and Intelligent System Development Laboratory in the University of China Academy of Sciences. This dataset contains two categories of targets: vehicles, aircraft. Some details of the dataset are shown in Table 4. All the targets labelled as vehicles are used in the evaluation process.

**Table 4:** UCAS-AOD dataset categories information.

Class name	Version1.0	Version2.0	Total
Plane	3591	3891	7482
Vehicle	4475	2639	7114

#### 4.1.3. VEDAI datasets

The VEDAI dataset is proposed in [18] in 2015. The images of the VEDAI dataset were taken from the satellite in the spring of 2012. The authors manually selected 1210 1024×1024 images from immense original images. All of these images were taken at the same height and the same shooting angle from the sky. Selected images have a variety of backgrounds, e.g. fields, grass, mountains, urban area, etc. As shown in Table 5, the VEDAI dataset contains nine different categories of vehicles: car, pick-up, truck, plane, boat, camping car, tractor, van, and others. These categories are divided into two meta-classes. The “small land vehicles” class consists of the “car”,”pick-up”,”tractor” and”van” categories. The “large land vehicles” class consists of the “truck” and”camping car” categories. In this paper, all the objects from the “small land vehicles” meta-category are used in the evaluation process.

**Table 5:** VEDAI dataset categories information.

Class name	Total	Meta-class name	Total
Pickup	950	Small Land Vehicles	2950
Tractor	190		
Vans	100		
Car	1340		
Truck	300	Large Land Vehicles	690
Camping Car	390		
Plane	47	-	-
Others	200		
Boat	170		

#### 4.1.4. DOTA datasets

The DOTA dataset is proposed in [20] in 2017. Most images in DOTA-v1.5 are collected from Google Earth, while the others are taken by Satellite JL-1 and Satellite GF-2. The resolutions of images contained in the DOTA dataset various from 800×800 to 4000×4000. As shown in Table 6, the dataset is labelled with 16 categories, namely “plan”, “ship”, “storage tank”, “baseball diamond”, “tennis court”, “basketball court”, “ground track field”, “harbor”, “bridge”, “large, small vehicle“,”helicopter“,”roundabout“, “soccer ball field” and “swimming pool”. In previously published researches of vehicle detection in aerial images, the DOTA dataset is mainly used in three different ways. Some algorithms, e.g. [21, 38], are evaluated using targets form “small vehicle” only. Some algorithms, e.g. [39, 40], are evaluated using targets from both “small vehicle” and “large vehicle”. Others, e.g. [11, 41], are evaluated using targets from “small vehicle”, “large vehicle”, “plane”, and “boat”.

**Table 6:** DOTA dataset categories information.

Class name	Total
Large-vehicle	11552
Small-vehicle	12841
Harbor	5723
Ship	23467
Ground track field	398
Soccer ball field	407
Baseball diamond	495
Swimming pool	986
Roundabout	417
Tennis court	2044
Basketball	353
Plane	6629
Helicopter	196
Bridge	1227
Storage tank	7636

Since targets in some categories, e.g. “baseball diamond”, “tennis court”, “basketball court”, “harbor”, “bridge”, are irrelevant to the theme of this paper, only the targets labelled as “small vehicle”, “ship” and “plane” are used in this partition to evaluate the performance of the proposed algorithm on targets with various scales. In these three categories, small vehicles have relatively stable scales (from 2×9 to 89×124), planes have various scales (from 9×8 to 856×842) but distinctive shapes, while the scales of ships various rapidly(from 5×12 to 939×1750).

### 4.2. Training detail and preprocessing on dataset

The proposed FFDP-CNN is an end to end one stage algorithm. It is implemented on the TensorFlow 2.0 deep learning framework, trained and evaluated using NVIDIA GeForce RTX 2080Ti with 11G memory. The weight decay and the momentum are set to 1*e*^−4^ and 0.9 respectively. In the experiments, the learning rate is set to 1*e*^−3^ initially, and gradually decades to 1*e*^−4^ and 1*e*^−5^.

Images from the three datasets are pre-processed before fed into the network. Through the sliding window, padding, and stride mechanism, all datasets are processed into images with a resolution of 640×640. Besides, the annotations of all datasets are optimized. The optimization process corrects the vehicle labels if targets are obviously missing. This is a common dataset optimization method that is also used in [21, 27].

### 4.3. Evaluation method

To evaluate the performance of the proposed framework, Average Precision (AP),mean Average Precision (mAP) and F1 are used in the experiments.

#### 4.3.1. P-R curve, AP and mAP

The main purpose of object detection is to find out targets and classify them into various categories. The evaluation indicators for these two tasks are "the proportion of the correct targets detected to all targets" and "the correct rate of the classification of detected targets", which can be represented by recall(abbreviated as R) and precision(abbreviated as P). The these two measurements are defined using true positive(TP), false positive (FP), true negative (TN), and false negative (FN).

The precision is calculated as shown in formula (7):

$$P=\frac{TP}{TP+FP},$$ (7)

The recall is calculated as shown in formula (8):

$$R=\frac{TP}{TP+FN},$$ (8)

When comparing the performance of different frameworks, the precision and recall indicators sometimes appear contradictory. Moreover, it is necessary to measure the accuracy of a detector with a single index. Therefore, AP(average precision) is proposed in [34]. P and R are sorted according to score, and draw a rectangular coordinate graph with P and R as the coordinates. This rectangular graph is called P-R curve. The area under the P-R curve is AP, and mAP (mean average precision) is the average of APs of multiple categories. AP measures the quality of the detector in each category, while mAP measures the quality of the detector in all categories. As a result, in this paper, AP is used when targets are from only one category (i.e. evaluations using UCAS-AOD and VEDIA datasets), and mAP is used when targets are from multiple categories (i.e. evaluation using DOTA dataset).

#### 4.3.2. F-Measure

F-Measure (also known as F-Score) is another commonly used indicator for object detectors. The F-Measure is a weighted average of Precision(P) and Recall(R):

$$F=\frac{(\alpha^{2}+1)P\times R}{\alpha^{2}(P+R)},$$ (9)

When α equal 1, F-Measure can be called F1:

$$F=\frac{2\times P\times R}{P+R},$$ (10)

F1 combines the results of P and R. when F1 is higher, it means that the detector is more effective. The P and R values of the proposed framework on various datasets are also provided for comprehensive evaluation.

### 4.4. Results on UCAS-AOD dataset

**Fig 6:**
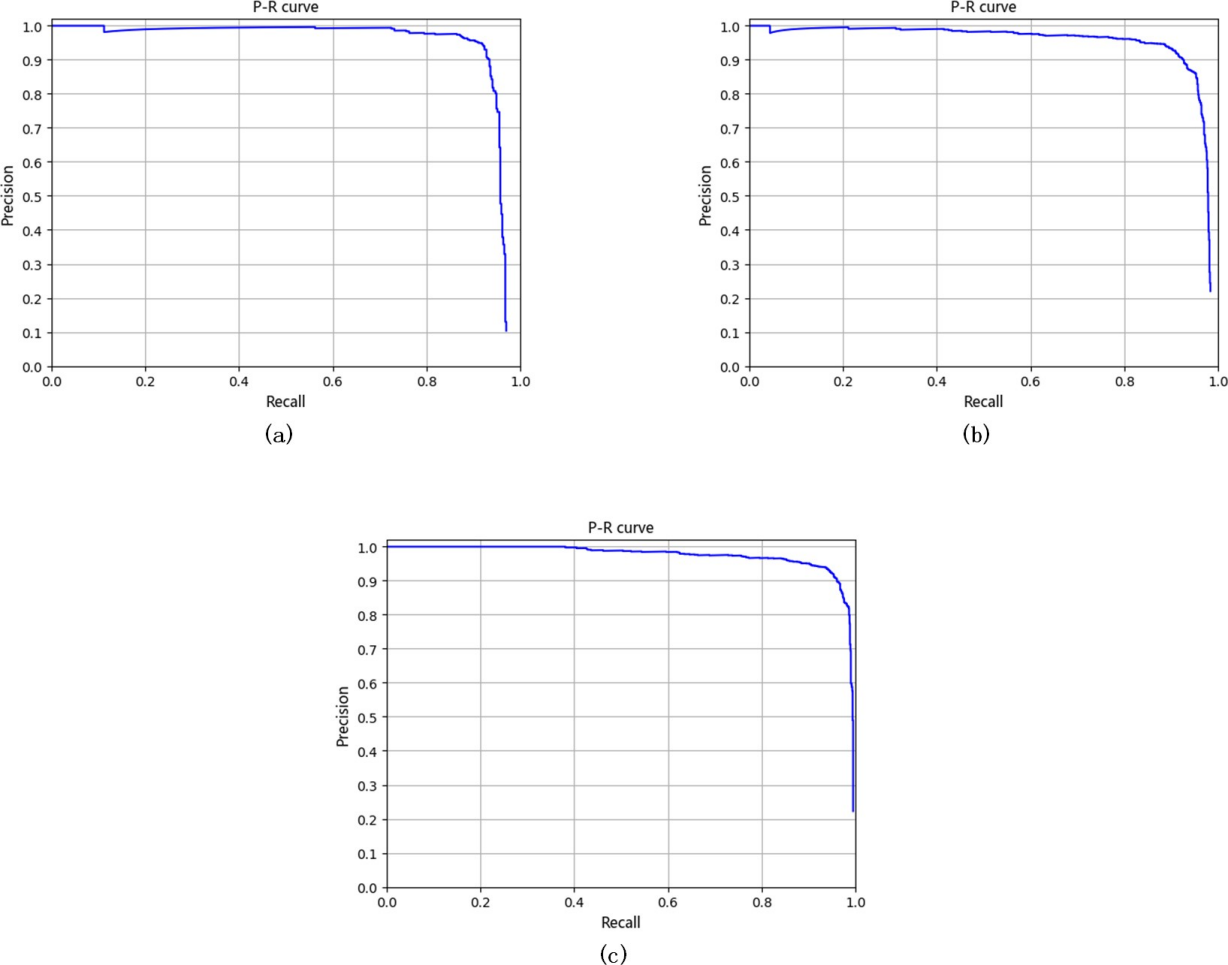
(a) P-R curve of FPN with Stepwise Res-block (b) P-R curve of FFDP-CNN with Res2Net (c) P-R curve of FFDP-CNN.

The effectiveness of the proposed framework has been validated on the UCAS-AOD dataset. The APs of the proposed FFDP-CNN detector and other 18 state-of-the-art vehicle detection algorithms for aerial images are shown in Table 7. It can be seen that the proposed FFDP-CNN detector achieves an AP of 97.34% which is roughly 1.2% higher than the APs achieved by UCAS + NWPU + VS-GANs 2019 [42] and Improved FBPN-Based Detection Network [38]. The P-R curve and the detailed performance of the proposed FFDP-CNN detector are shown in Fig 6 and Tables 7, 8 respectively.

**Table 7:** Comparison between proposed framework and other methods on UCAS-AOD.

Method	AP(%)
YOLO v2 2017 [43]	79.20
SSD 2020 [16]	81.37
R-DFPN 2018 [44]	82.50
Improved Faster RCNN 2017 [27]	83
DRBox 2017 [45]	85
*O*^2^-DNet 2016 [46]	86.72
P-RSDet 2020 [47]	87.36
R-FCN 2016 [48]	89.3
Deformable R-FCN 2017 [49]	91.7
S2ARN 2019 [50]	92.2
FADet 2019 [51]	92.72
RetinaNet-H 2019 [52]	93.6
R3Det 2019 [52]	94.14
A2RMNet 2019 [27]	94.65
SCRDet + + 2020 [53]	94.97
ICN 2018 [54]	95.67
UCAS + NWPU + VS-GANs 2019 [42]	96.12
Improved FBPN-Based Detection Network [38]	96.18
**FPN with Stepwise Res-block**	**94.86**
**FFDP-CNN with Res2Net**	**95.38**
**FFDP-CNN**	**97.34**

**Table 8:** Comparison between proposed framework and other methods on UCAS-AOD.

Method	R	P	AP(%)	F1-Score
**FPN with Stepwise Res-block**	**91.9**	**91.0**	**94.86**	**91.48**
**FFDP-CNN with Res2Net**	**93.4**	**91.1**	**95.38**	**92.27**
**FFDP-CNN**	**93.8**	**93.1**	**97.34**	**93.42**

To demonstrate the effectiveness of the proposed stepwise res-block and feature fusion deep-projection module, two other algorithms (FFDP-CNN with Res2Net and FPN with Stepwise Res-block) have been evaluated, and the evaluation results are shown in Table 8.

#### 4.4.1 Effectiveness of stepwise res-block

Compared with the proposed FFDP-CNN (which utilizes Stepwise Res-block and FFDP), FFDP-CNN with Res2Net utilizes Res2Net blocks to build its backbone while other parts remain the same with FFDP-CNN. In other words, the only difference between FFDP-CNN and FFDP-CNN with Res2Net is the res-block used in their backbone. As a result, the effectiveness of the proposed Stepwise Res-block can be demonstrated by comparing the evaluation results of FFDP-CNN and FFDP-CNN with Res2Net. According to the evaluation results shown in Table 8, FFDP-CNN with Res2Net achieves an AP of 95.38% which is 1.96% lower than the AP obtained by FFDP-CNN. In other words, comparing with Res2Net, Stepwise res-block gains an advantage of 1.96% AP in the evaluation.

#### 4.4.2 Effectiveness of feature fusion deep projection module

Comparing with FFDP-CNN, FPN with Stepwise Res-block utilizes FPN instead of feature fusion and deep-projection module (FFDP), while other parts remain the same. As a result, the effectiveness of the proposed FFDP can be demonstrated by comparing the evaluation results of FFDP-CNN and FPN with Stepwise Res-block. From Table 8, it can be seen that compared with standard FPN, the feature fusion deep-projection module (FFDP) achieves a solid performance advantage of 2.48% AP.

#### 4.4.3 Parameter number and processing speed

To demonstrate the efficiency of the proposed stepwise res-block, the parameter number of the proposed FFDP-CNN and other algorithms are shown in Table 9. It can be seen that FFDP-CNN has less parameter comparing with other state-of-the-art algorithms (i.e. YOLO, Faster RCNN and An Improved FBPN). Because of the complexity of the proposed feature fusion and deep-projection module, the processing speed of the proposed FFDP-CNN for 640×640 color images is 14.01 FPS on an NVIDIA 2080Ti GPU.

**Table 9:** Comparison of parameter numbers and other information.

Method	Input size	Model size	Parameter Number
YOLO V2 [11]	608×608	255 MB	67 M
YOLO V3 [11]	608×608	235 MB	61 M
Faster R-CNN [11]	608×608	253 MB	59 M
RetinaNet [11]	608×608	146 MB	36 M
AVDNet [11]	608×608	53 MB	13 M
An Improved FBPN [38]	600×600	479 MB	61 M
Ju.M et.al [21]	512×512	2.8 MB	-
**FFDP-CNN**	**640×640**	**144 MB**	**37 M**

#### 4.4.4 Performance analysis

The detection results of the proposed FFDP-CNN are analyzed manually to get a comprehensive understanding of its performance. In the detection results, most targets can be found correctly regardless of their orientations, colours and scales, which also demonstrated the effectiveness of the proposed framework. However, the proposed algorithm sometimes fails when parts of the targets are blocked by trees or building roofs in the input images, especially when the blocked parts are more than half of the original targets.

### 4.5. Results on VEDAI dataset

**Fig 7:**
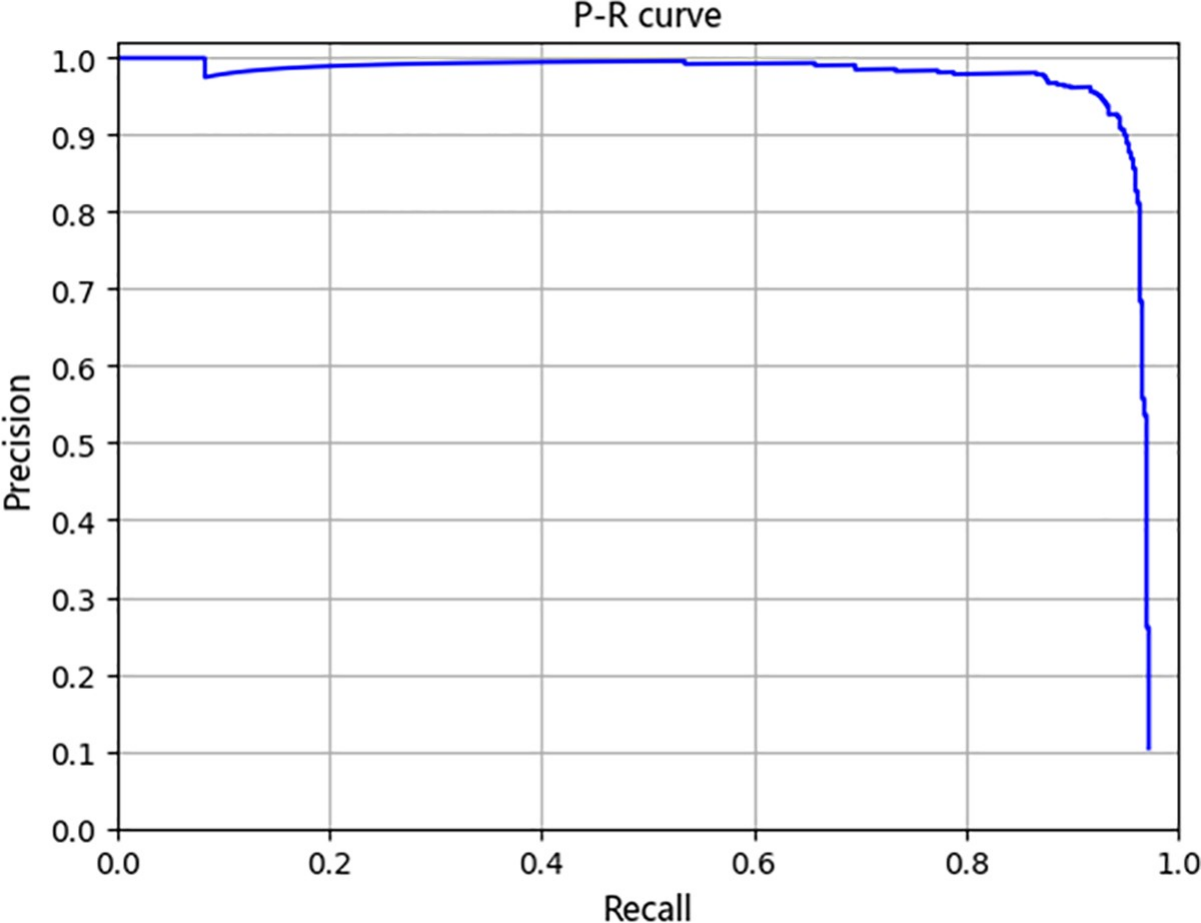
The P-R curve of our framework on VEDAI.

The proposed FFDP-CNN is trained and evaluated on objects labelled as “small land vehicles” in VEDAI dataset. The APs of the proposed detector and other 13 state-of-the-art algorithms are shown in Table 10. The proposed FFDP-CNN detector achieves an AP of 93.96%, outperforms other state-of-the-art networks designed for vehicle detection. Compared with DFL [11], the AP has been improved by 3.42%. Compared with Improved FBPN-based Detection Network [38], the AP has been improved by 2.69%. The P-R curve and the detailed performance of the proposed FFDP-CNN detector are shown in Fig 7 and Table 11 respectively.

**Table 10:** Comparison between proposed framework and other methods on VEDAI.

Method	AP(%)
AVDNet 2019 [11]	51.95
VDN 2017 [41]	54.6
DPM 2015 [55]	60.5
*R*^3^-Net (R + F) 2019 [56]	69.0
Faster-RCNN 2017 [55]	70.9
Improved Faster RCNN 2017 [43]	74.30
Ju, et al. 2019 [21]	80.16
YOLOv3_Joint-SRVDNet 2020 [57]	80.4
Faster RER-CNN 2018 [55]	83.5
YOLOv3_HR [57]	85.66
Faster RCNN + Res2Net (resnet101) 2019 [58]	81.96
Faster RCNN + WaterFall (resnet50) 2019 [28]	77.36
Improved FBPN-Based Detection Network [38]	91.27
**FFDP-CNN**	**93.96**

**Table 11:** F1-Measure on VEDAI.

Method	R	P	AP(%)	F1-Score
**FFDP-CNN**	**94.9**	**90.5**	**93.96**	**92.67**

### 4.6. Results on DOTA dataset

**Fig 8:**
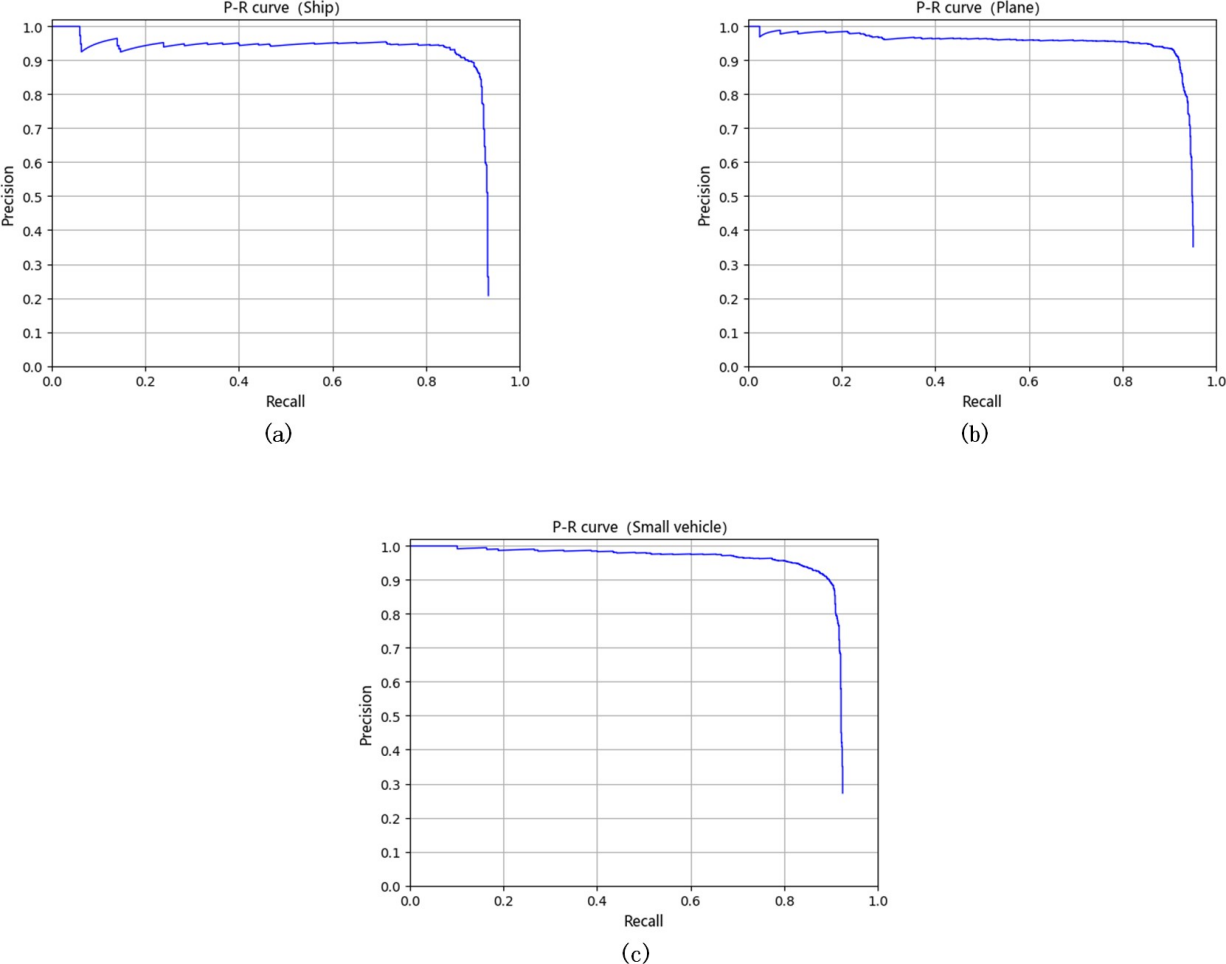
The P-R curve of our framework on DOTA different categories, (a) is the P-R curve on Ship, (b) is the P-R curve on Plane, (c) is the P-R curve on Small Vehicle.

The proposed framework has also been evaluated by the DOTA dataset. The evaluation results of the proposed FFDP-CNN detector and other 16 state-of-the-art algorithms are shown in Table 12. In general, it can be seen that the proposed framework achieves an mAP of 89.86% which is higher than the mAPs obtained by other state-of-the-art algorithms. The P-R curves of the proposed FFDP-CNN algorithm based on the DOTA dataset are shown in Fig 8. Table 13 demonstrates the detailed evaluation results of the proposed framework. Table 12 also demonstrate that the proposed FFDP-CNN achieves the best performance when detecting small vehicles (with an AP of 89.78%), and the second-best performance when detecting planes and ships (with APs of 91.33% and 88.48% respectively). As a result, comparing to other algorithms, the performance of FFDP-CNN can maintain at a relatively stable level when the scales of the targets various rapidly.

**Table 12:** Comparison between proposed framework and other methods on DOTA.

Method	AP(%) (Ship)	AP(%) (Plane)	AP(%) (Small Vehicle)	mAP(%)
L-RCNN 2020 [39]	-	-	56.09	-
Yang et al. 2018 [38]	-	-	61.16	-
RoITransformer [59]	83.59	88.64	68.81	80.35
the Light-Head R-CNN OBB+W/FPN [59]	75.77	88.02	70.15	77.98
Faster RCNN Adapted 2018 [60]	87.7	87.4	74.9	83.33
DYOLO Module B 2018 [60]	**88.7**	88.2	76.0	84.3
SSD Adapted2018 [60]	85.0	88.2	76.3	83.17
DFRCNN 2018 [61]	-	-	76.5	-
DSSD 2017 [62]	87.5	91.1	79.0	85.87
DYOLO Module A 2018 [60]	87.8	86.6	79.2	84.53
RefineDet 2018 [60]	87.5	**94.0**	80.0	87.17
Ju, et al. 2019 [21]	-	-	88.63	-
Improved FBPN-Based Detection Network [38]	-	-	88.76	-
**FFDP-CNN**	88.48	91.33	**89.78**	**89.86**

**Table 13:** F1-Measure on DOTA.

Method	R	P	mAP(%)	F1-Score
**FFDP-CNN on Ship**	**87.37**	**91.42**	**88.48**	**89.35**
**FFDP-CNN on Plane**	**90.94**	**93.07**	**91.33**	**91.99**
**FFDP-CNN on Small Vehicle**	**89.0**	**91.1**	**89.78**	**90.0**

## 5. Conclusion and future works

In this paper, a Feature Fusion Deep-Projection Convolution Neural Network (FFDP-CNN) is proposed to detect vehicles in aerial images. The main contribution of this research has three aspects. Firstly, a novel residual block named stepwise res-block is designed in this paper. Thanks to its special hierarchical structure, the output of the stepwise res-block contains features processed by 0–3 convolutional layers. Besides, the parameter number keeps at a relatively low level. Secondly, based on the proposed stepwise res-block, the backbone of the proposed FFDP-CNN is designed. By composing 34 stepwise res-blocks, the proposed backbone produce features processed by 0–102 3x3 convolutional layers. It can explore the high-level semantic feature and conserve the low-level detail feature at the same time. Last but not least, a feature fusion module is used to mix features generated from different levels of the backbone, and further balance the low-level and high-level features. A special designed deep-projection deconvolution module is utilized in the proposed feature fusion module to reduce the impact of information contamination introduced by downsampling/upsampling processes. According to the evaluation results, the proposed FFDP-CNN outperforms other state-of-the-art algorithms on UCAS-AOD, VEDAI and DOTA datasets. According to the detection results, although the proposed FFDP-CNN can detect targets regardless of their orientations, colours and scales, the ability to detect partially blocked targets still can be improved. In the future work, GANs will be used to generate more partially blocked training data to train the proposed framework to detect partially blocked targets, and attention mechanisms will be implemented in the stepwise res-block to further increase the detection accuracy.
